# Next-Generation Sequencing Identifies Deregulation of MicroRNAs Involved in Both Innate and Adaptive Immune Response in ALK+ ALCL

**DOI:** 10.1371/journal.pone.0117780

**Published:** 2015-02-17

**Authors:** Julia Steinhilber, Michael Bonin, Michael Walter, Falko Fend, Irina Bonzheim, Leticia Quintanilla-Martinez

**Affiliations:** 1 Institute of Pathology and Neuropathology, Tübingen, Germany; 2 Comprehensive Cancer Center, Tübingen, Germany; 3 Department of Medical Genetics and Applied Genomics, MFT Servives, University Hospital Tübingen, Eberhard-Karls-University, Tübingen, Germany; University of Hong Kong, HONG KONG

## Abstract

Anaplastic large cell lymphoma (ALCL) is divided into two systemic diseases according to the expression of the anaplastic lymphoma kinase (ALK). We investigated the differential expression of miRNAs between ALK+ ALCL, ALK- ALCL cells and normal T-cells using next generation sequencing (NGS). In addition, a C/EBPβ-dependent miRNA profile was generated. The data were validated in primary ALCL cases. NGS identified 106 miRNAs significantly differentially expressed between ALK+ and ALK- ALCL and 228 between ALK+ ALCL and normal T-cells. We identified a signature of 56 miRNAs distinguishing ALK+ ALCL, ALK- ALCL and T-cells. The top candidates significant differentially expressed between ALK+ and ALK- ALCL included 5 upregulated miRNAs: miR-340, miR-203, miR-135b, miR-182, miR-183; and 7 downregulated: miR-196b, miR-155, miR-146a, miR-424, miR-503, miR-424*, miR-542-3p. The miR-17-92 cluster was also upregulated in ALK+ cells. Additionally, we identified a signature of 3 miRNAs significantly regulated by the transcription factor C/EBPβ, which is specifically overexpressed in ALK+ ALCL, including the miR-181 family. Of interest, miR-181a, which regulates T-cell differentiation and modulates TCR signalling strength, was significantly downregulated in ALK+ ALCL cases. In summary, our data reveal a miRNA signature linking ALK+ ALCL to a deregulated immune response and may reflect the abnormal TCR antigen expression known in ALK+ ALCL.

## Introduction

Anaplastic large cell lymphoma (ALCL) represents a distinct group of T-cell non-Hodgkin lymphomas, which are separated according to the World Health Organization (WHO) classification [1] into two different disease entities based on the presence or absence of a chromosomal translocation involving the anaplastic lymphoma kinase (*ALK*) gene. ALK+ ALCL is characterized, in most cases, by the t(2;5)(p23;q35) chromosomal translocation involving the nucleophosmin (*NPM*) and the *ALK* gene, resulting in the expression and constitutive activation of chimeric ALK fusion protein. The oncogenic NPM-ALK with its transforming ability activates several downstream signaling pathways, mainly RAS/MAPK, PLCγ, PI3K and JAK/STAT pathways, which participate in cell proliferation, differentiation and survival [2,3,4,5,6,7].

One central downstream target of ALK is the transcription factor CCAAT/enhancer binding protein beta (C/EBPβ) [8,9,10,11]. C/EBPβ is involved in a number of cellular processes, including differentiation, proliferation, inflammatory responses and metabolism [12,13]. Moreover it has been associated with tumorigenesis in solid tumors [14,15] and plays an important role in ALK+ ALCL oncogenesis [8,10,16]. We recently reported that C/EBPβ in ALK+ ALCL mediates important functions such as cell proliferation and survival by transcriptional activation of its target genes [16]. Besides its functions in transcriptional gene regulation, C/EBPβ is able to regulate target gene expression also posttranscriptionally via miRNA induction [17,18,19].

miRNAs are a noncoding class of 17–24 base single-stranded RNA molecules that are able to posttranscriptionally regulate their target genes by either mRNA degradation or translational repression, and have become a major focus of research in molecular biology [20,21,22,23,24]. miRNAs are involved in many important biological processes including the immune response, different stages of hematopoietic development, and the regulation of cellular differentiation and apoptosis [25,26,27]. Deregulated miRNAs are able to drive oncogenesis acting either as tumor suppressors or oncogenes [28].

Two recent studies have aimed to characterize the miRNA signature associated with ALCL to identify new downstream effectors of the ALK oncogenic pathway [29,30]. Merkel et al. [29] demonstrated that members of the miR-17-92 cluster, which have been associated with inhibition of apoptosis, promotion of proliferation and induction of tumor angiogenesis are highly expressed in ALK+ ALCL, whereas miR-155, which is involved in the immune response and has oncogenic potential, was expressed at higher levels in ALK- ALCL. Using a high throughput TaqMan quantitative real-time PCR (RT-qPCR) approach in primary ALCL cases, Liu et al. [31] corroborated the high expression of the miR-17-92 cluster in ALK+ ALCL and found a signature of 7 additional miRNAs that could help to distinguish ALK+ from ALK- ALCL cases (5 upregulated: miR-512-3p, miR-886-5p, miR-886-3p, miR-708, miR-135b; 2 downregulated: miR-146a, miR-155). Interestingly, the miRNA signature of ALK-ALCL was found to have a different profile compared with peripheral T cell lymphoma (PTCL), not otherwise specified (NOS), and to partially overlap with the miRNA expression prolife of ALK+ ALCL, suggesting that the pathogenesis of ALK- ALCL is closer to ALK+ ALCL than to PTCL, NOS.

The so far reported posttranscriptional regulation potential of C/EBPβ in several systems raised the question to which extent C/EBPβ controls miRNA expression in ALK+ ALCL cells. Thus the aim of this study was to analyze the differential expression of miRNAs between ALK+ and ALK- ALCLs and ALK+ and normal T-cells, respectively, using next generation sequencing (NGS), and to generate in addition, a C/EBPβ-dependent miRNA profile. Our data reveal a signature that may link ALK+ ALCL to a deregulated immune response and may in part be responsible for the abnormal TCR antigen expression known in ALK+ ALCL.

## Materials and Methods

### Cell culture, virus production, viral infections and patient samples

The four ALK+ ALCL cell lines (SUDHL-1, KiJK, Karpas 299 and SR-786) and the ALK- ALCL cell line (Mac-1) were cultured as recently described [8,16]. The ALK+ ALCL cell lines (SUDHL-1, KiJK, Karpas 299 and SR-786) were provided by Mark Raffeld (National Cancer Institute, NIH, Bethesda, MD, USA), and cultured as recently described [8]. SUDHL-1, Karpas 299 and SR-786 were purchased from the American Type Culture Collection (ATCC) and KiJK was obtained from the author [32]. All four ALK+ ALCL cell lines have been authenticated and are suitable for in vitro model system for ALCL [33]. The ALK- ALCL cell line Mac-1 was provided by Eva Geißinger (University of Würzburg, Germany). Production of virus containing lentiviral vector pFUGW or pRRL.PPT.SF.i2GFPp [34] and viral infection of ALK+ ALCL cells to down-regulate C/EBPβ or up-regulate C/EBPβ isoforms LAP and LAP* was accomplished as previously specified [16,35,36,37]. After 72 h gene transduction efficiency was determined by flow cytometric analysis of GFP-positive cells as recently described [35].

Primary tumor samples of five ALK+ and four ALK- ALCLs and four reactive lymph nodes (RLN) were collected from the archives of the Institute of Pathology, University of Tübingen, Germany. All cases were systematically immunophenotyped, as part of the diagnostic work-up, and were classified following the recommendations of the WHO classification for tumors of haematopoietic and lymphoid tissues [38]. Macrodissection was performed in cases containing few tumor cells.

CD3+ T-cells were isolated from peripheral blood of healthy individuals using MACS Separation System (Miltenyi Biotec, CA, USA). Pre-enrichment of PBMCs was performed by standard Ficoll density gradient centrifugations. T-cells were labeled with CD3 MicroBeads (Miltenyi Biotec, CA, USA) and magnetically separated using VarioMACS.

Ethics approval for the study (620/2011BO2) was obtained from the Ethics Committee at the Medical Faculty, University Tübingen. Written informed consent was obtained for the lymphoma specimens and blood samples of healthy donors used in this study.

### Western blot analysis

Lysis of cells and Western blotting were performed as described elsewhere [9,35]. For immunoblotting we used the C/EBPβ C-19 antibody (1:200, Santa Cruz Biotechnology, Santa Cruz, CA, USA) and the ALK 400 antibody (1:1000, Life Technologies, Carlsbad, CA, USA) or the α-Tubulin antibody (1:5000, Sigma-Aldrich, Steinheim, Germany) as loading controls.

### RNA isolation

Total RNA was isolated using the RNeasy Mini Kit (Qiagen, Hilden, Germany), including DNase treatment in cell lines. Total RNA including miRNA was isolated using the miRNeasy Mini Kit (Qiagen, Hilden, Germany) in cell lines. Total RNA was extracted from formalin fixed paraffin embedded tissues in primary cases using phenol/chloroform extraction followed by DNase treatment (DNA-free Kit, Applied Biosystems, Ambion, Carlsbad, CA, USA), as described elsewhere [39].

### Next-generation sequencing

Library Preparation: Small RNA sequencing libraries were constructed from 1 μg total RNA using the TruSeq Small RNA Library Prep Kit (Illumina, San Diego, CA) according to the manufacturer’s instructions. Libraries were loaded onto an E-Gel (Invitrogen) and miRNA enriched fractions running at 147 nt were isolated, purified and quantified by fluorospectrometry on a Qbit instrument (Invitrogen). Equimolar amounts of all 12 libraries were pooled and loaded onto two lanes of an Illumina GAIIx single-read flow cell. Clusters were generated in a cBot instrument (Illumina) using TrueSeq Cluster Generation Kit (Illumina). A single 32 nt sequencing run was performed to obtain miRNA sequences followed by a 7 nt sequencing run to determine the barcode sequences. Data preprocessing: Raw sequence files were processed and de-multiplexed with CASAVA1.7 (Illumina) to obtain fastq files. Fastq ASCII qualities were transferred from Illumina v1.3 to Sanger scale with FastQ Groomer [40]. Linker sequences were removed with fastx clipper and reads with less than 15 nt were discarded (http://hannonlab.cshl.edu/fastx_toolkit/index.html). Mature miRNAs were identified by the web-based miRanalyzer tool, allowing up to 1 mismatch (http://bioinfo2.ugr.es/miRanalyzer/miRanalyzer.php) [41,42]. First, reads with identical sequences are grouped using a pearl script and subsequently compared against miRBase v16 to identify mature miRNAs in the read groups. NGS data were submitted to the European Nucleotide Archive with the study accession number PRJEB7797.

Statistical analysis: miRNAs with less than 5 counts in any sample were removed from the data set. Differentially expressed miRNAs were calculated using the Bioconductor package ‘DESeq’ (Anders and Huber Genome Biology 2010, 11:R106). miRNAs with a FDR of less than 0.05 were called differentially expressed.

### Real-time quantitative RT-PCR

RNA was transcribed into cDNA using Superscript II reverse transcriptase (Invitrogen, Carlsbad, CA, USA), a mix of Oligo(dT) primer (Promega, Madison, WI, USA) and random hexamers (Roche Applied Science, Penzberg, Germany) according to the manufacturer’s instructions. Real-time quantitative RT-PCR analysis (RT-qPCR) to quantify the mRNA levels of C/EBPβ using the TATA box-binding protein (TBP) as housekeeping gene, the LightCycler 480 Probes Master and the LightCycler 480 System for detection (Roche Applied Science, Penzberg, Germany), was carried out as previously described [43].

For mature miRNA quantification, cDNA was synthesized from 500 nanograms of miRNA applying the miScript Reverse Transcription Kit (Qiagen, Hilden, Germany). miRNAs were quantified using miScript Primer assays Hs_miR-203_1, Hs_miR-146b_1, Hs_miR-181a*_1, Hs_miR-181a_2, Hs_miR-181c_2, Hs_miR-29c_1, Hs_miR-342_1, Hs_miR-146a_1, Hs_miR-182_2, Hs_miR-183_2, Hs_miR-7_2, Hs_miR-486_1 (Qiagen, Hilden, Germany). PCR was performed with the miScript SYBR Green PCR Kit (Qiagen, Hilden, Germany) using 2 μl diluted cDNA in a 20 μl final reaction mixture (15 min at 95°C, 55 cycles of 15 seconds at 95°C, 30 seconds at 55°C, 30 seconds at 70°C, melting curve at 60–97°C with the specific product at 74–77°C).

miRNA expression was normalized to RNU6B or miR-106b using miScript Primer assays Hs_RNU6–2_1, Hs_miR-106b_1 (Qiagen, Hilden, Germany), since miR-106b was consistently expressed among the used cell lines, tumor and control samples.

Data were analyzed using the 2^-ΔΔCp^ method as previously described [9]. All reactions were done in duplicates.

## Results

### Deep sequencing identifies the small RNA transcriptome of ALCL cells and CD3+ PBMCs

Deep sequencing was performed on small RNA libraries generated from triplicates of three ALK+ ALCL cell lines (SUDHL-1, KiJK, Karpas 299) after C/EBPβ silencing or from mock-treated cells, one ALK- ALCL cell line (Mac-1) and from CD3+ PBMCs from healthy donors. Altogether 41.8 Gb of sequencing data were generated. Sequencing and filtering of low-quality sequences of these 24 samples small RNA fractions yielded from 3 to 7.5 million sequence reads in the samples respectively (Table 1). After analysis using miRanalyzer [44,45] from 10% to 33% of the reads were identified as miRNAs (miRBase v16). In total, deep sequencing revealed 789 unique miRNA sequences whereupon for the respective individual sample 420–535 miRNAs were detected.

**Table 1:** Numbers of reads and identified miRNAs obtained by deep sequencing.

Sample	Cluster raw	number of reads passed chastity filter	% of reads passed chastity filter	number of miRNA counts	% miRNA counts of all filtered reads	number of expressed miRNAs
SUDHL-1 pF 1	3248094	3025885 +/- 59861	93.16 +/- 1.84	303836	10.04	422
SUDHL-1 pF 2	5427105	5121416 +/- 98948	94.37 +/- 1.82	1000842	19.54	474
SUDHL-1 pF 3	5854238	5574723 +/- 97016	95.23 +/- 1.66	1524934	27.35	507
SUDHL-1 pF-C/EBPβ 1	5527464	5226932 +/- 101784	94.56 +/- 1.84	663549	12.69	479
SUDHL-1 pF-C/EBPβ 2	6512519	6187778 +/- 117407	95.01 +/- 1.80	885793	14.32	496
SUDHL-1 pF-C/EBPβ 3	5105264	4817850 +/- 97143	94.37 +/- 1.90	552989	11.48	471
KiJK pF 1	6551387	6189796 +/- 125248	94.48 +/- 1.91	1392231	22.49	466
KiJK pF 2	6409664	6070191 +/- 119601	94.70 +/- 1.87	1202191	19.80	460
KiJK pF 3	6233591	5774569 +/- 100938	92.64 +/- 1.62	902376	15.63	446
KiJK pF-C/EBPβ 1	6944042	6558613 +/- 134612	94.45 +/- 1.94	740369	11.29	420
KiJK pF-C/EBPβ 2	5188620	4939483 +/- 95251	95.20 +/- 1.84	670372	13.57	429
KiJK pF-C/EBPβ 3	6955221	6572955 +/- 134026	94.50 +/- 1.93	681570	10.37	434
Karpas 299 pF 1	5040603	4745420 +/- 92662	94.14 +/- 1.84	994856	20.96	488
Karpas 299 pF 2	6368833	6008816 +/- 113786	94.35 +/- 1.79	885832	14.74	490
Karpas 299 pF 3	6412193	6115285 +/- 107126	95.37 +/- 1.67	1639117	26.80	535
Karpas 299 pF-C/EBPβ 1	5494884	5208200 +/- 98689	94.78 +/- 1.80	1020257	19.59	478
Karpas 299 pF-C/EBPβ 2	5992929	5700842 +/- 105082	95.13 +/- 1.75	1054658	18.50	473
Karpas 299 pF-C/EBPβ 3	5050189	4777934 +/- 93234	94.61 +/- 1.85	961111	20.12	482
Mac-1 1	5548500	5250492 +/- 103022	94.63 +/- 1.86	1414087	26.93	513
Mac-1 2	7967308	7527369 +/- 145580	94.48 +/- 1.83	1224189	16.26	503
Mac-1 3	6394602	5957192 +/- 99504	93.16 +/- 1.56	1002338	16.83	501
T cells 1	6026769	5712751 +/- 109498	94.79 +/- 1.82	1881343	32.93	483
T cells 2	6166769	5878655 +/- 105977	95.33 +/- 1.72	1913121	32.54	502
T cells 3	5775300	5470379 +/- 105932	94.72 +/- 1.83	1462615	26.74	483

Numbers of unfiltered and filtered reads, percentage of filtered reads and numbers of miRNA reads with indication of percentage as well as numbers of different miRNAs identified are depicted for each sample applied to deep sequencing.

### Principal Component Analysis (PCA) of miRNA deep sequencing data

**Fig 1:**
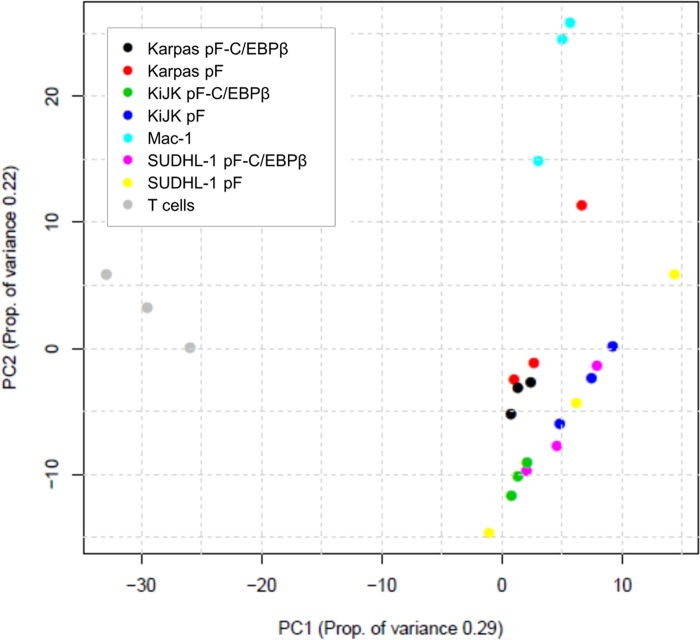
Principal Component Analysis. 2D scatter plot shows principal component analysis (PCA) of miRNA deep sequencing data. The two axes represent the first two principal components (PCs) from the principal component analysis. The values in brackets indicate the amount of variation in the data that can be explained by the PC. The percent of variation given by a particular PC is indicated in the axis label. Points are colored by sample type. Samples were analyzed in triplicates. The graph shows a clear separation by principal component 1 between normal T cells (grey) and ALCL cells. The principal component 2 separates ALK+ and ALK- ALCL (light blue).

In order to illustrate the overall variation of the samples, miRNA sequencing data was analyzed by PCA, which graphically converts and reduces the multidimensionality of expression profiles into a 2D scatter plot. miRNA expression data from triplicates of three ALK+ ALCL cell lines (SUDHL-1, KiJK, Karpas 299) after C/EBPβ silencing or from mock-treated cells, one ALK- ALCL cell line (Mac-1) and from CD3+ PBMCs from healthy donors (T cells) was subjected to PCA (Fig. 1). The data shows a clear separation between normal T cells and ALCL cells, both with ALK+ and ALK-. The first component separates T cells and ALCL cells. The second component (PC2) clusters the different ALK+ ALCL cell lines and separates them from the ALK- ALCL cell line.

### Differentially expressed patterns of miRNAs between ALK+ ALCL, ALK- ALCL and normal T cells

**Fig 2:**
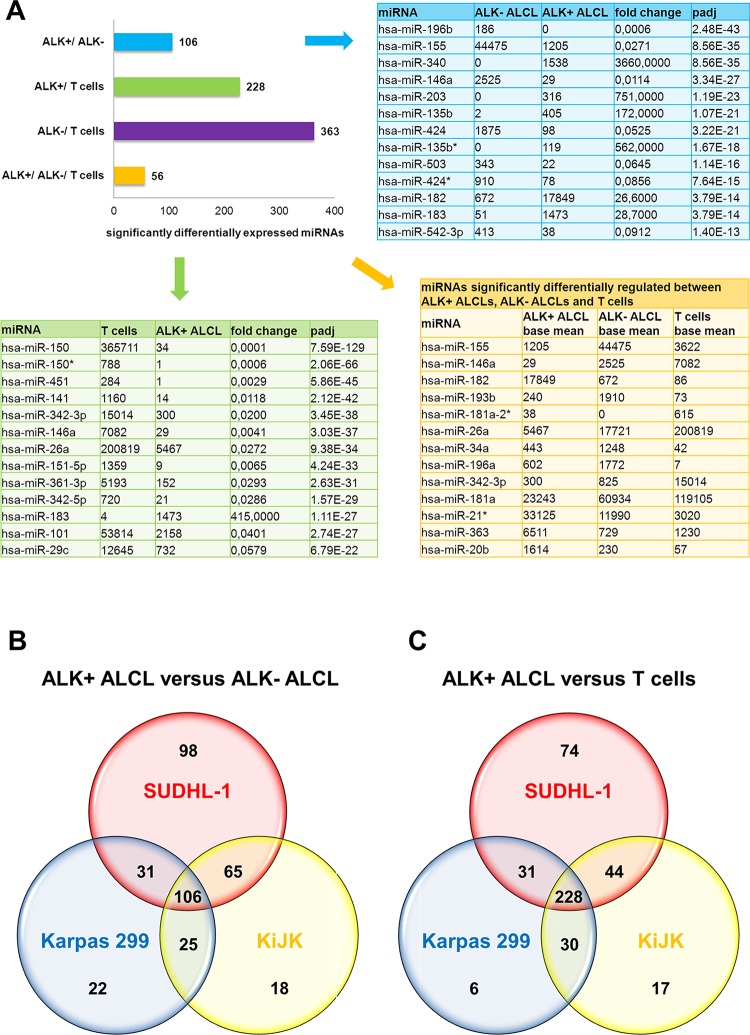
Differentially expressed miRNAs in ALK+, ALK- ALCL and normal T cells. (**A**) The bar graph illustrates the numbers of significantly differentially expressed miRNAs between the combined three ALK+ ALCL cell lines SUDHL-1, KiJK and Karpas 299, the ALK- ALCL cell line Mac-1 and normal T cells. The 13 most significantly differentially expressed miRNAs between the investigated ALK- ALCL cell line and the three ALK+ ALCL cell lines (upper right table) and the 13 miRNAs most significantly differentially expressed between the investigated three ALK+ ALCL cell lines and normal T cells (left table) are additionally designated. Data in both tables are depicted as base means of triplicates and fold change and Benjamini-Hochberg corrected p-values (padj) are indicated. Lower right table lists the top 13 miRNAs, which are significant differentially expressed in ALK+ ALCL, ALK- ALCL and normal T cells and present highest differences in expression levels. (**B**) The Venn diagram represents significant differentially expressed miRNAs in ALK+ and ALK- ALCL in the three ALK+ ALCL cell lines SUDHL-1, KiJK and Karpas 299. The analysis reveals a common profile of 106 deregulated miRNAs. (**C**) The Venn diagram illustrates significant differentially expressed miRNAs in ALK+ ALCL and T cells in the three ALK+ ALCL cell lines SUDHL-1, KiJK and Karpas 299. The analysis shows a common profile of 228 deregulated miRNAs.

**Fig 3:**
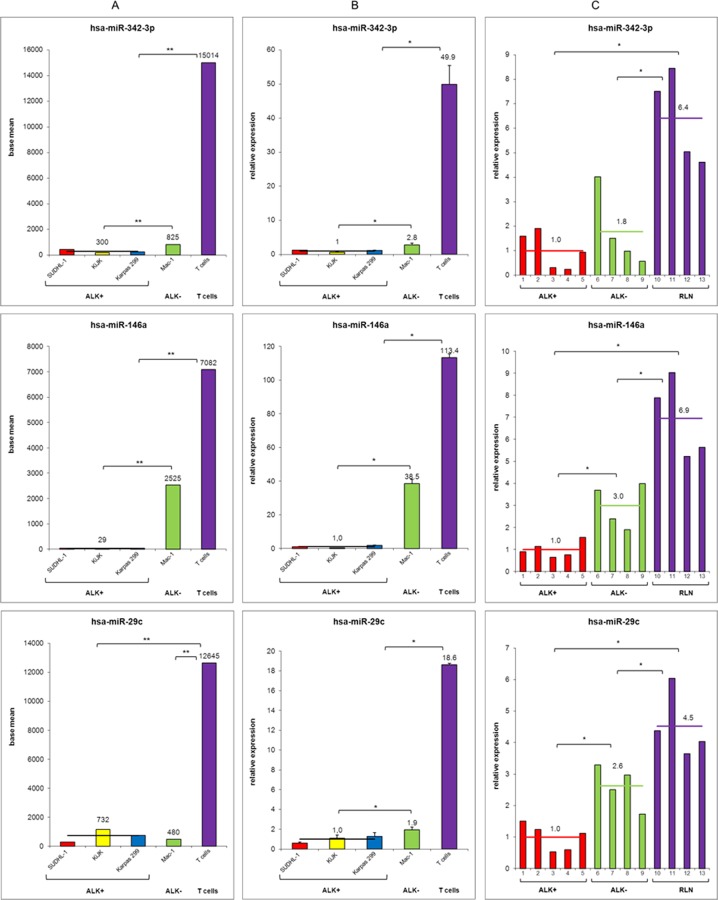
Comparative relative miRNA expression of miRNAs miR-342-3p, miR-146a and miR-29c—deep sequencing, RT-qPCR of cell lines and primary samples. Quantification of three differentially expressed miRNAs miR-342-3p (upper panel), miR-146a (middle panel) and miR-29c (lower panel) between ALK+ ALCL, ALK- ALCL, T cells (isolated from 3 healthy donors), and normal lymph nodes, respectively. (**A**) Deep sequencing results. Data are depicted as base mean values from triplicates. (**B**) RT-qPCR validation analysis. Error bars indicate standard deviation of triplicates for cell lines and number of tested samples, respectively. (**C**) RT-qPCR validation analysis in primary ALCL cases and reactive lymph nodes (RLN). For RT-qPCR quantification values were normalized to miR-106b and data were analyzed according to the 2^-ΔΔCp^ method. Results are depicted as miRNA levels relative to mean value ALK+ ALCL levels. For statistical analysis of RT-qPCR results a Wilcoxon rank-sum test was used (*p<0.05, **p<0.01).

We performed miRNA expression profiling analysis evaluating the relative expression of the detected miRNAs (miRBase v16). In order to identify specifically deregulated miRNAs in ALK-driven ALCL, we compared ALK+ (SUDHL-1, KiJK and Karpas 299) versus ALK- ALCL (Mac-1), and versus normal T cells (Fig. 2A). All miRNAs with a padj-value (Benjamini-Hochberg corrected p-value) of less than 0.05 were called significant differentially expressed. The miRNAs were selected only according to the adjusted p-value. We found 106 differentially expressed miRNAs between ALK+ and ALK- ALCL (Fig. 2B), and 228 miRNAs between ALK+ ALCL and normal T cells (Fig. 2C). Eighty-two of these miRNAs were differentially expressed between ALK+ and ALK- ALCL cells, and between ALK+ ALCL and T cells (S1 Table). The top 13 miRNAs candidates in both groups are depicted in Fig. 2A. The comparison between Mac-1 and normal T cells revealed 363 miRNAs differentially expressed. In total 56 miRNAs were significantly differentially expressed in the three groups, providing a distinct expression profile characteristic for ALK+ and ALK- ALCL, and T cells (S1 Table). To restrict this expression profile, the top 13 miRNAs showing significant differences in the expression levels between ALK+, ALK- ALCL and T cells were selected (Fig. 2A). To corroborate the sequencing results regulated miRNAs were validated by RT-qPCR (S2 Table). Three regulated miRNAs (miR-342–3p, miR-146a and miR-29c), which showed high expression levels in T cells and very low expression levels in ALCL cells were selected for validation by RT-qPCR in the cell lines and in primary ALCL cases (5 ALK+ and 4 ALK- ALCL). As control 4 normal lymph nodes were analyzed (Fig. 3A-C). The expression pattern of all three miRNAs was confirmed by RT-qPCR in the cell lines (Fig. 3B) and in the primary cases (Fig. 3C). In summary, validation experiments confirmed the reproducibility of the results obtained by deep sequencing (S2 Table). The three miRNAs (miR-342–3p, miR-146a and miR-29c) analyzed showed strong deregulation in ALK+ ALCL compared to normal T cells (Fig. 3A). However, only miR-146a and miR-29c showed significantly different expression levels between ALK+ and ALK- ALCL cases suggesting an important role of these two miRNAs in the pathogenesis of ALK+ ALCL. The levels of expression in ALK- ALCL cases were generally intermediate between the ALK+ ALCL cases and the normal lymph nodes used as controls (Fig. 3C).

### Identification of miRNAs significantly regulated by C/EBPβ

**Fig 4:**
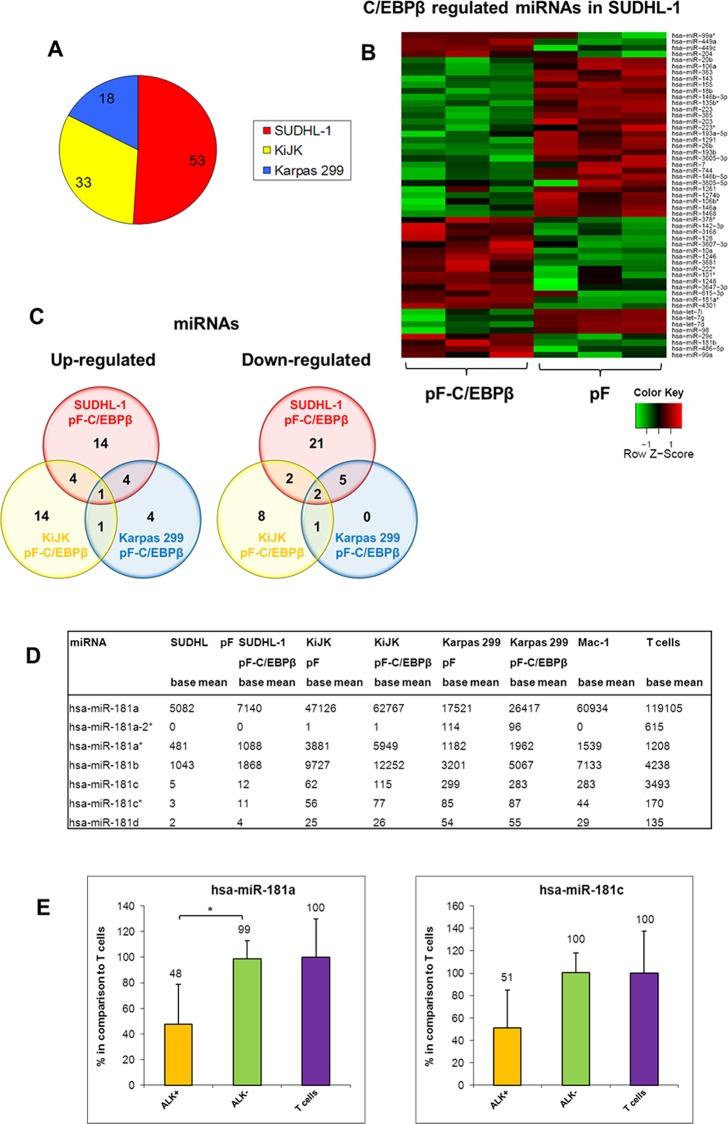
MiRNAs significantly regulated by C/EBPβ knockdown in ALK+ ALCL cell lines. MiRNA expression profiling of the three ALK+ ALCL cell lines SUDHL-1, KiJK and Karpas 299 with (pF-C/EBPβ) and without (pF) C/EBPβ down-regulation. (**A**) The circle diagram indicates the number of miRNAs regulated by C/EBPβ knockdown in the particular ALK+ ALCL cell line. (**B**) C/EBPβ-signature of SUDHL-1 cells derived by C/EBPβ-shRNA. Heatmap shows the expression pattern of the 53 miRNAs significantly regulated by C/EBPβ in SUDHL-1 cells, depicting transduced cells with C/EBPβ-shRNA and controls in triplicates. (**C**) The Venn diagrams illustrate the number of significantly down-regulated (left) and up-regulated (right) miRNAs in the ALK+ ALCL cell lines after transduction with C/EBPβ-shRNA. (**D**) The table shows from all miR-181 family members the miRNA expression levels (base mean of triplicates) of the three ALK+ ALCL cell lines SUDHL-1, KiJK and Karpas 299 with (pF-C/EBPβ) and without (pF) C/EBPβ knockdown as well as the ALK- ALCL cell line Mac-1 and normal T cells. (**E**) RT-qPCR analysis of miR-181a and miR-181c expression in ALK+ (SUDHL-1, KiJK and Karpas 299) and ALK- (Mac-1) ALCL cell lines and T cells. Error bars indicate standard deviation of triplicates for cell lines and number of tested samples, respectively. For RT-qPCR quantification values were normalized to miR-106b and data were analysed according to the 2^-ΔΔCp^ method. Results are depicted as miRNA levels relative to mean value of T cell levels. For statistical analysis of RT-qPCR results a Wilcoxon rank-sum test was used (*p<0.05).

In order to identify the miRNAs, which are regulated by C/EBPβ, we performed next-generation sequencing of miRNAs of the three ALK+ ALCL cell lines SUDHL-1, KiJK and Karpas 299 after successful C/EBPβ down-regulation. (S1 Fig.) [8,16]. Statistical analysis of the identified miRNAs resulted in 80 different miRNAs significantly regulated by C/EBPβ (Fig. 4). In SUDHL-1 cells, we found 53 miRNAs (23 up- and 30 down-regulated after C/EBPβ down-regulation) (Fig. 4A+B), in KiJK 33 miRNAs (20 up- and 13 down-regulated after C/EBPβ knockdown) and in Karpas 299 18 miRNAs (10 up- and 8 down-regulated after C/EBPβ down-regulation) significantly regulated by C/EBPβ (S3 Table, Fig. 4A). The decreasing number of regulated miRNAs follows the same pattern as C/EBPβ expression observed in previous studies in these cell lines [16]. Forty-two miRNAs were significantly up-regulated in at least one of the three ALK+ ALCL cell lines after C/EBPβ knockdown (Fig. 4C). Nine of these miRNAs (miR-181b, miR-1246, miR-1248, miR-3168, miR-486-5p, miR-29c, miR-3607-3p, miR-4301 and miR-3182) were significantly higher expressed in two cell lines while one miRNA (miR-181a*) was significantly up-regulated in all three ALK+ ALCL cell lines after C/EBPβ down-regulation. Because of the important role of the miR-181 family in the immune response, all family members were investigated (Fig. 4D). All 7 members of the miR-181 family showed very low expression in ALK+ ALCL cells when compared to ALK- ALCL cells and normal T cells. Two family members (miR-181a and miR-181c) were additionally validated via RT-qPCR in all three ALK+ ALCL cell lines (Fig. 4E). Although there was a clear difference in expression between ALK+ and ALK- ALCL cells, only miR-181a showed a statistically significant difference suggesting a role of miR-181a in the pathogenesis of ALK+ ALCL. Among the down-regulated miRNAs after C/EBPβ knockdown, 39 miRNAs were significantly regulated in at least one of the three ALK+ ALCL cell lines. Eight of them (miR-1291, miR-143, miR-193b, miR-365, miR-744, miR-345, miR-146b-3p and miR-7) were significantly decreased in two cell lines and two miRNAs (miR-146b-5p and miR-203) were down-regulated in all three analysed ALK+ ALCL cell lines. Taken together, we found 3 miRNAs significantly regulated by C/EBPβ in all three investigated ALK+ ALCL cell lines.

**Fig 5:**
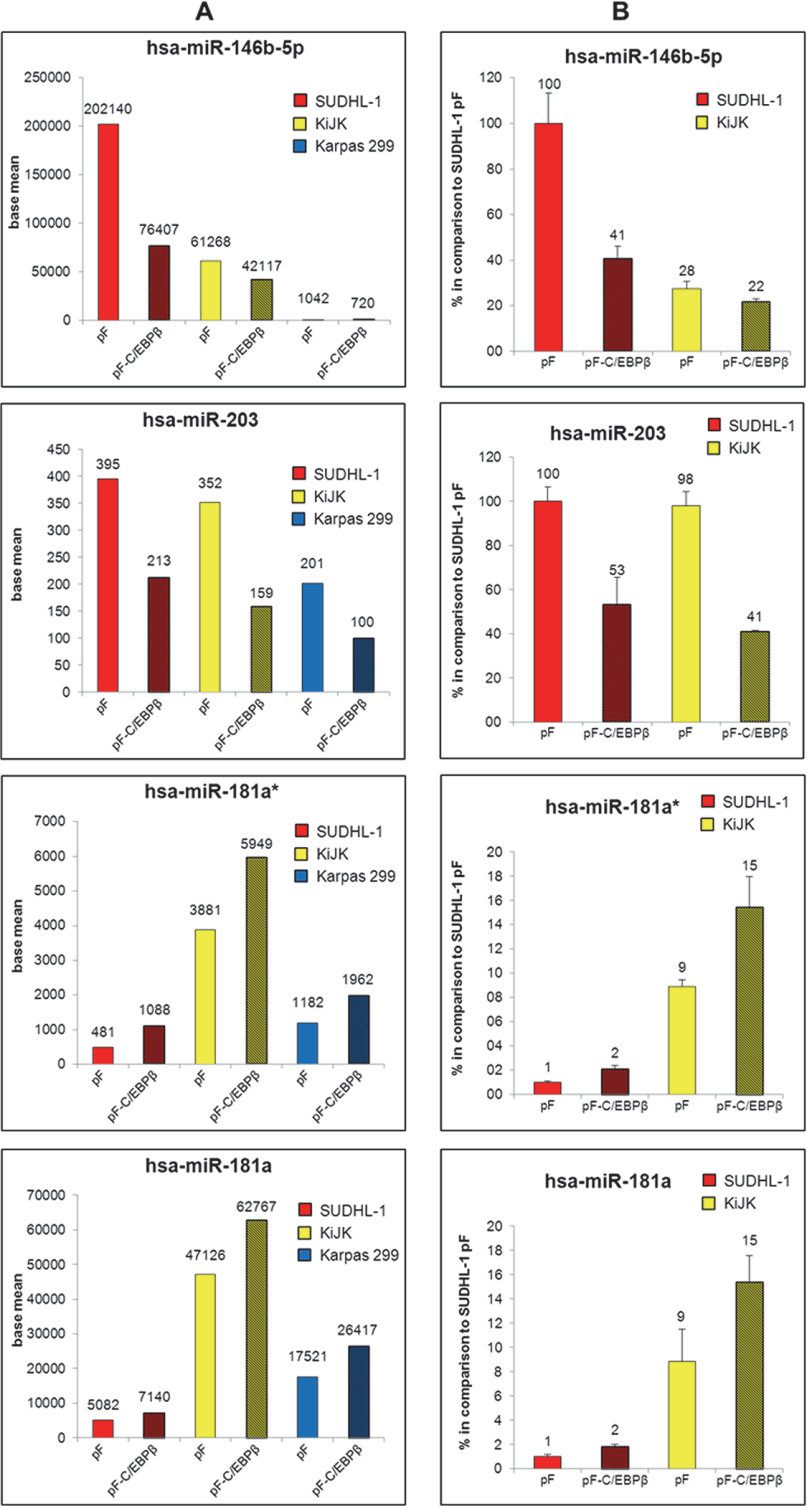
Comparative relative miRNA expression of miRNAs miR-146b-5p, miR-203, miR-181a* and miR-181a after C/EBPβ down-regulation—deep sequencing and RT-qPCR of ALK+ ALCL cell lines. Quantification of three by C/EBPβ knockdown significantly regulated miRNAs miR-146b-5p (upper panel), miR-203 (second panel), miR-181a* (third panel) and additionally miR-181a (lower panel), in ALK+ ALCL cell lines. (**A**) Deep sequencing results of ALK+ ALCL cell lines transduced with pF or pF-C/EBPβ shRNA. Results are depicted as base mean values from triplicates. (**B**) RT-qPCR analysis of miRNAs 146b-5p, 203, 181a* and 181a in pF and pF-C/EBPβ (shaded) transduced ALK+ ALCL cells four days after infection. Values were normalized to RNU6B and data were analysed according to the 2^-ΔΔCp^ method. Results are depicted as miRNA levels relative to untreated SUDHL-1 cells. Error bars indicate standard deviation of triplicates.

**Fig 6:**
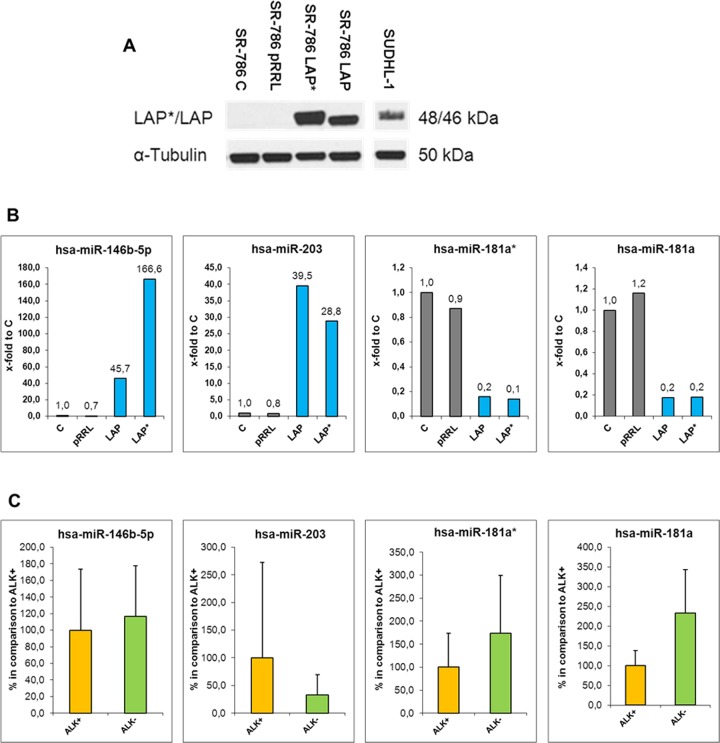
Analysis of C/EBPβ regulated miRNAs. (**A**) Western Blot analysis of the C/EBPβ isoforms LAP* and LAP in the transduced SR786 cells three days after infection. Each lane of the Western Blot contains 20 μg protein extract. Tubulin was used as loading control. SR786 = uninfected cells, pRRL = empty virus, pRRL-LAP* = virus containing the C/EBPβ isoform LAP* sequence, pRRL-LAP = virus containing the C/EBPβ isoform LAP sequence. (**B**) RT-qPCR analysis of miRNAs 146b-5p, 203, 181a* and 181a in untreated, mock-treated and pRRL.PPT.SF.i2GFPp (containing LAP and LAP* isoforms) transduced ALK+ ALCL cell line SR-786. Values were normalized to miR-106b and data were analysed according to the 2^-ΔΔCp^ method. Results are depicted as miRNA levels relative to untreated SR-786 cells. (**C**) RT-qPCR analysis of miRNAs 146b-5p, 203, 181a* and 181a in primary ALCL cases (4 ALK+ and 5 ALK- ALCL cases). For RT-qPCR quantification values were normalized to miR-106b and data were analysed according to the 2^-ΔΔCp^ method. Results are depicted as miRNA levels relative to mean value of ALK+ ALCL levels. For statistical analysis of RT-qPCR results a Wilcoxon rank-sum test was used (*p<0.05).

These three miRNAs (miR-181a*, miR-146b-5p and miR-203) were validated via RT-qPCR analysis in SUDHL-1 and KiJK cells (Fig. 5A-B). In addition, the expression levels were compared to normal T cells (S2 Table). Normal T cells showed the highest expression levels of miR-181a and the lowest levels of miR-203 and miR-146b-5p. To further confirm C/EBPβ-dependent miRNA expression, the isoforms LAP* and LAP, which have an activating transcriptional function, were overexpressed in the ALK+ ALCL cell line SR786, which does not express C/EBPβ (Fig. 6A). Strong upregulation of C/EBPβ isoforms LAP and LAP* corroborated C/EBPβ dependent regulation of all four analysed miRNAs (Fig. 6B). As expected, miR-181a* and miR-181a were down-regulated whereas miRNAs 146b-5p and 203 were clearly up-regulated after LAP and LAP* overexpression compared to controls. The expression of these four miRNAs was further validated in the primary ALCL cases (Fig. 6C). miR-181a* and miR-181a were confirmed to be downregulated in ALK+ ALCL when compared with ALK- ALCL primary cases whereas miR-203 was found to be upregulated in ALK+ ALCL when compared to ALK- ALCL primary cases. We did not find a difference in miR-146b-5p expression between ALK+ and ALK- ALCL primary cases. In summary, miR-181a*, miR-181a and miR-203 are transcriptionally regulated by C/EBPβ in ALK+ ALCL cell lines and primary cases and most probably play an important role in the pathogenesis of the disease.

## Discussion

We previously reported that in ALK+ ALCL, ALK protein induces the expression of the transcription factor C/EBPβ primarily through STAT3, and that C/EBPβ plays a central role in ALK-mediated oncogenesis [29,31]. In the current study, in order to identify the miRNAs specifically expressed in ALK+ ALCL, and possibly regulated by C/EBPβ, we performed miRNA NGS of ALK+ and ALK- cell lines, normal T cells and ALK+ ALCL cell lines after C/EBPβ silencing. PCA analysis of the sequencing data confirmed characteristic patterns among samples of different entities and with downregulated C/EBPβ expression, confirming the use of this approach to characterize the miRNA expression pattern in ALCL. We show that the miRNA signature of ALK- ALCL has a different profile compared with normal T cells, and to partially overlap with the miRNA expression prolife of ALK+ ALCL, indicating that the two ALCL subgroups are closely related.

Processing of NGS data generated miRNA expression profiles and identified 228 and 106 miRNAs significantly differentially expressed between ALK+ ALCL and T cells or ALK+ ALCL and ALK- ALCL cells, respectively. ALK- ALCL cells and T cells showed a significant difference in the expression levels of 366 miRNAs. These data provided a signature of 56 miRNAs with a specific expression profile characteristic for ALK+ and ALK- ALCLs and T cells. A striking hundred-fold change in expression levels was observed for some miRNAs of the different signatures, which may contribute to certain characteristics of these diseases. Thirteen miRNAs with high differences in expression levels between ALK+, ALK- ALCL and T cells provide a limited, probably biologically relevant miRNA profile for ALK+ ALCLs (Fig. 2A).

Comparison of our data with two previously published miRNA profiles of ALK+ ALCL provided further validation of our results [29,31]. Not surprisingly, using NGS we found more significantly differentially expressed miRNAs between the different entities than it was feasible using miRNA arrays [29] or a high throughput TaqMan quantitative real-time PCR (qRT-PCR) approach [31]. Although there are considerable differences mainly due to the technical approach and material used (cell lines versus primary cases), our miRNA signature shows overlapping results with 26 miRNAs identified by Merkel et al. [29] and Liu et al. [31] (S4 Table). Of these, nine miRNAs were found regulated or preferentially associated with ALK+ ALCL in all three studies (miR-106a, miR-20b, miR-363, miR-17, miR-93, miR-101, miR-20a, miR-146a and miR-155). The overlapping detection of deregulated miRNAs in the different studies indicates that at least some of them most probably contribute to ALK mediated oncogenesis and/or tumor biology. The low expression of miR-146a and miR-155, both on the top of the list of differentially expressed miRNAs in our study, as a constant feature in ALK+ ALCL, is intriguing because evidence suggests that these two miRNAs may have crucial roles in regulating the innate immune response and that the putative targets of both miRNAs are components of the toll-like receptor (TLR) signaling machinery [20,25].

miR-146a is a member of the miR-146 miRNA family consisting of two evolutionary conserved miRNA genes; *miR-146a* and *miR-146b* [46]. They share the same seed sequence but are encoded by different loci in the genome. miR-146a expression occurs in an NF-κB (nuclear factor κB) dependent manner in response to LPS (Lipopolysaccharides). Recent studies have suggested that miR-146a acts as a negative feedback regulator of the innate immune response by targeting two adapter proteins TNF-receptor-associated factor 6 (TRAF6) and IL-1 receptor-associated kinase 1 (IRAK1) that are crucial for proinflammatory signaling and activation of NF-κB [46]. In contrast, lack of miR-146a expression results in exaggerated inflammatory response and spontaneous autoimmune disorders [47]. Aging miR-146a deficient mice developed myeloid sarcomas and lymphomas associated with chronic NF-κB activation, which suggests that miR-146a may also function as a tumor suppressor gene. The excessive inflammation seen in these mice argues in favor of a connection between chronic inflammation and cancer. Most interesting is the fact that miR-146a expression might be involved in cell fate determination in T cells because its expression is associated with T cell expansion and a T-helper (Th) 1 phenotype with strong TCR stimulation, and low expression induces a Th2 differentiation [48]. Furthermore, miR-155 deficient mice, in addition to having a defect in the germinal center reaction, show a skewing of their T cells toward Th2 differentiation with low production of interferon γ [49]. Accordingly, our analysis of the cytokine expression in cell lines (data not shown) demonstrated that ALK+ ALCL cells are characterized by very high expression of IL-10 with very low expression of interferon γ. Therefore, it is tempting to speculate that the low expression of miR-146a and miR-155 might influence the unique immunophenotype of the ALK+ ALCL tumor cells and the tumor microenvironment [50]. Interestingly, miR-135b, which was one of the most strongly ALK-upregulated miRNAs in our study and in the study of Liu et al. [31], was recently confirmed to be differentially expressed between ALK+ and ALK- ALCL [51]. miR-26a, miR-29c and miR-150 are interesting candidates because of their very low expression in ALK+ ALCL, as compared to normal T cells. The downregulation of all three miRNAs have been found to promote proliferation and favor migration and metastasis [52]. miR-150 is important for the differentiation of T cells and has been suggested to act as a tumor suppressor in T-cell lymphomas [53]. miR-29c is one of the four members of the miR-29 family, which has been reported to contribute to several cellular processes such as apoptosis, cell proliferation, extracellular matrix regulation, differentiation and immune response [54]. We confirmed also the down-regulation of miR-29a in ALK+ ALCL cell lines, which was recently shown to upregulate the antiapoptotic protein MCL1 contributing to tumor cell survival [29,55]. Interestingly miR-29a and miR-29c were significantly up-regulated after C/EBPβ knockdown, indicating that they are not only regulated downstream of ALK but also downstream of C/EBPβ.

Accordingly, another major aim of this study was to investigate which of the miRNAs differentially expressed in ALK+ ALCL might be direct or indirect targets of the transcription factor C/EBPβ. C/EBPβ has been shown to influence the expression of miRNAs by activation or inhibition of miRNA transcription. Fittingly, we found 80 miRNAs significantly regulated by C/EBPβ in the investigated ALK+ ALCL cell lines. Four miRNAs (miR-181a*, miR-181a, miR-146b-5p and miR-203) were validated in all three ALK+ ALCL cell lines but only three of them were found to be differentially expressed in ALK+ ALCL primary cases (miR-181a*, miR-181a, miR-203). Of special interest is the downregulation of the miR-181 family members by C/EBPβ. C/EBPβ is a key transcription factor regulating monocytic gene expression and thereby involved in the innate immune response [56]. In contrast, miR-181a is involved in the regulation of the adaptive immune response. miR-181a regulates T cell differentiation and influences T cell sensitivity to antigens by modulating TCR signalling strength controlling the expression of multiple phosphatases in the TCR signalling pathway. Decreased expression of miR-181a blocks T-cell differentiation and results in hyporesponsiveness to TCR signalling and decrease in sensitivity to antigens [57]. In a recent study, it was demonstrated that downregulation of miR-181a was extremely relevant to HTLV1 biology, and an important strategy of the virus to dampen TCR signalling and T-cell activation to persist in the host [58]. Similarly miR-181c was found to be downregulated by Hepatitis C virus (HCV) in chronic liver disease by modulating the expression of C/EBPβ [59]. Accordingly, the previously demonstrated lack of TCR signaling in ALCL [50] might result from the decreased expression of miR-181a/miR-181a* in ALK+ ALCL and provide a mechanism to evade immune surveillance. The low expression level of several miRNAs in ALK+ ALCL compared to the high expression level in normal T cells could be the reflection of the abnormal TCR signaling in ALCL.

Another interesting direct target of C/EBPβ was miR-203, which was significantly upregulated in all three ALK+ ALCL cell lines. miR-203 is not expressed in normal T cells [60,61] and was not expressed in the ALK- ALCL cell line Mac-1 in our study. Although its function remains elusive, miR-203 seems to have also a role in the immune response through regulation of the Suppressor of Cytokine Signaling-3 (SOCS-3) [60].

**Fig 7:**
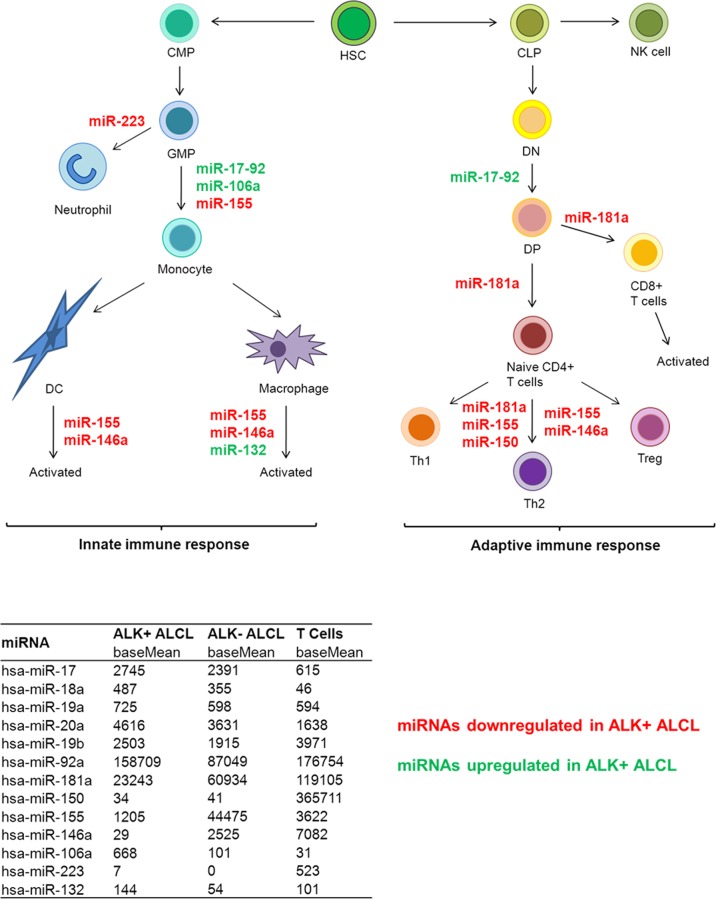
Deregulated miRNAs involved in the immune response in ALK+ALCL. MiRNAs with known functions in the immune response [62] and deregulated in ALK+ ALCLs in our study are shown (red: downregulated miRNAs in ALK+ ALCL, green: upregulated miRNAs in ALK+ ALCL). The miR-17~92 cluster comprises the 6 miRNAs 17, 18a, 19a, 20a, 19b and 92a. The table shows the deep sequencing results of these miRNAs as base mean values from triplicates. CLP, common lymphocyte progenitor; CMP, common myeloid progenitor; DC, dentritic cell; DP, double positive T cell; DN, double negative T cell; GMP, granulocyte monocytic progenitor; HSC, hematopoietic stem cell; MDP, myeloid dendritic progenitor.

In summary, using NGS we were able to identify miRNA expression profiles distinguishing ALK+, ALK- ALCL and normal T cells. We found that miRNAs involved in the differentiation and regulation of T cells of both the innate and adaptive immune response and inflammation are profoundly deregulated in ALK+ ALCL cell lines and primary cases (Fig. 7) [62]. Not surprisingly given the central role of C/EBPβ in the innate immune response, some of these miRNAs were found to be direct C/EBPβ targets. The deregulation of miR-181a together with miR-155, miR-150 and miR-146a might explain the lack of TCR signaling in ALCL cells. The potential role of miRNAs in the modulation of tumor immunophenotype and microenvironment offers an exciting hypothesis to understand the biology of ALK+ ALCL.

## Supporting Information

S1 Fig Lentiviral down-regulation of C/EBPβ in three ALK+ ALCL cell lines. The results of C/EBPβ knockdown are illustrated for the three ALK+ ALCL cell lines SUDHL-1, KiJK and Karpas 299. (**A**) Flow cytometry analysis of transduced SUDHL-1, KiJK and Karpas 299 cells with C/EBPβ shRNA (pF-C/EBPβ) or empty vector (pF) and control cells three days after infection. The percentage of GFP-positive cells represents the infected cells. (**B**) RT-qPCR analysis of C/EBPβ mRNA in SUDHL-1, KiJK and Karpas 299 cells four days after infection. Values were normalized to *TBP* and data were analyzed according to the 2^-ΔΔCp^ method. Results are represented as mRNA levels relative to control. Error bars indicate standard deviation of infected triplicates. (**C**) Western Blot analysis of C/EBPβ in the three transduced ALK+ ALCL cell lines four days after infection demonstrates successful knockdown. Each lane contained 30 μg protein extract. ALK was used as loading control.
(TIF)

S1 Table Significantly regulated miRNAs between ALK+ ALCLs, ALK- ALCLs and normal T cells. The 82 significantly regulated miRNAs between ALK+ ALCL cell lines and the ALK- cell line and between ALK+ ALCL cell lines and normal T cells are shown and expression (base mean) and significance (padj) values of the significantly regulated miRNAs are indicated. The 56 miRNAs additionally significantly regulated between ALK- ALCL cells and T cells are highlighted in grey.
(PDF)

S2 Table RT-qPCR validation of several miRNAs differentially regulated obtained by NGS. The expression level tendencies of twelve miRNAs were validated in the three ALK+ ALCL cell lines SUDHL-1, KiJK and Karpas 299, the ALK- ALCL cell line Mac-1 and T cells using RT-qPCR. RT-qPCR values were normalized to miR-106b and data were analyzed according to the 2^-ΔΔCp^ method. NGS results are represented as base mean expression and RT-qPCR results are shown as percentages relative to SUDHL-1 cells (100%).
(PDF)

S3 Table Significantly regulated miRNAs after C/EBPβ knockdown. The 80 significantly regulated miRNAs in at least one of the analyzed ALK+ ALCL cell lines by C/EBPβ are shown and miRNA expression levels (base mean of triplicates) of the three ALK+ ALCL cell lines SUDHL-1, KiJK and Karpas 299 with (pF-C/EBPβ) and without (pF) C/EBPβ knockdown as well as the ALK- ALCL cell line Mac-1 and normal T cells are depicted.
(PDF)

S4 Table Comparison of significantly ALK+ ALCL associated miRNAs between different studies [29,31]. Comparison of our data with two previously published miRNA profiles of ALK+ ALCL by Merkel et al. [29] and Liu et al. [31]. Shown are the miRNAs, which were found in at least two different studies associated with ALK+ ALCLs. In the studies different cell lines and/ or tumor specimens were used. X indicates, that miRNAs are differentially regulated by ALK and x* symbolizes, that miRNAs are associated with ALK+ ALCL.
(PDF)
