# Supplementary figures and images for: Next-Generation Sequencing Identifies Deregulation of MicroRNAs Involved in Both Innate and Adaptive Immune Response in ALK+ ALCL
Source: PLoS One. 2015 Feb 17;10(2):e0117780. doi: 10.1371/journal.pone.0117780 (PMC4331429; doi:10.1371/journal.pone.0117780)

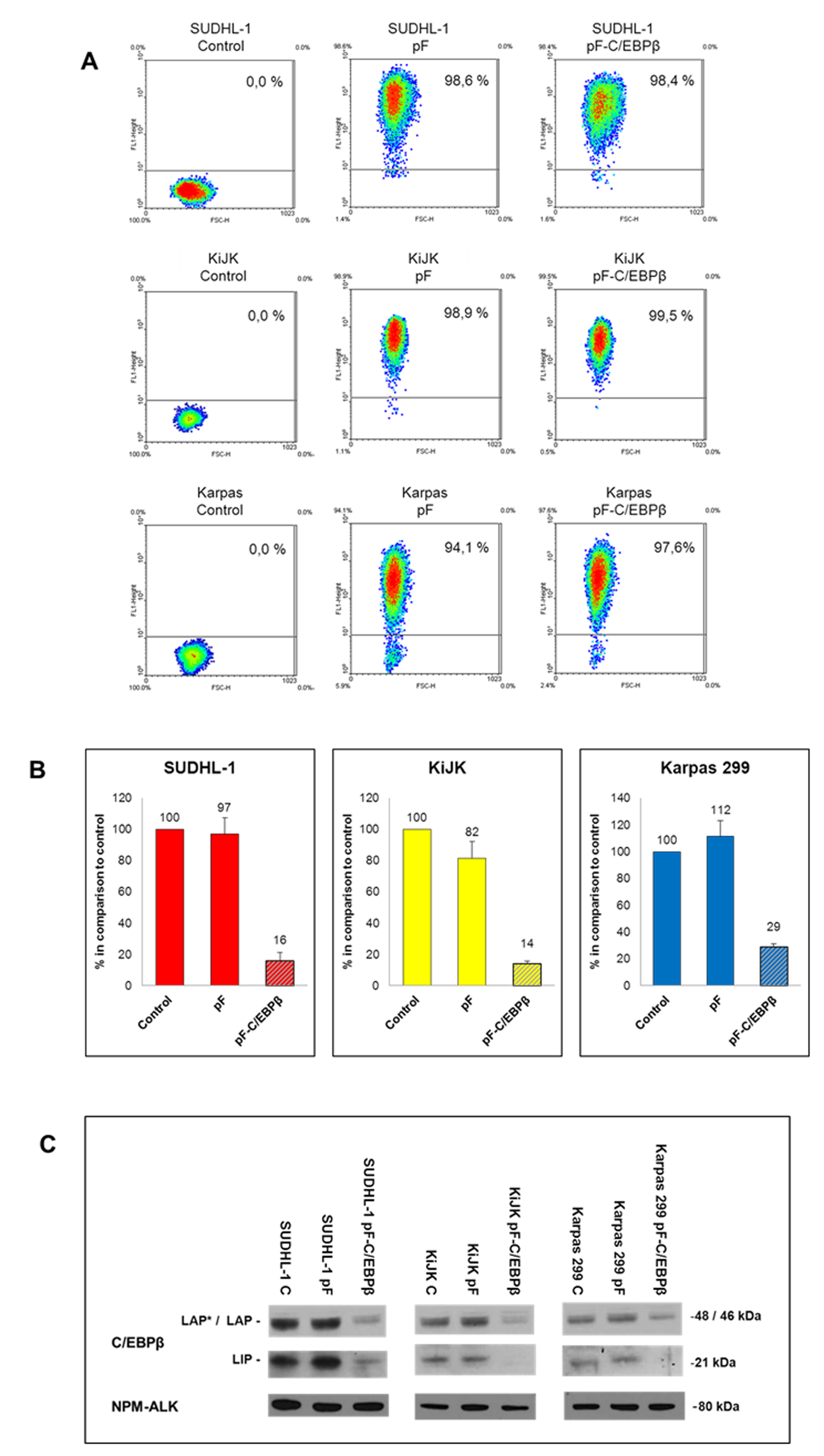

Supplement: S1 Fig — The results of C/EBPβ knockdown are illustrated for the three ALK+ ALCL cell lines SUDHL-1, KiJK and Karpas 299. (A) Flow cytometry analysis of transduced SUDHL-1, KiJK and Karpas 299 cells with C/EBPβ shRNA (pF-C/EBPβ) or empty vector (pF) and control cells three days after infection. The percentage of GFP-positive cells represents the infected cells. (B) RT-qPCR analysis of C/EBPβ mRNA in SUDHL-1, KiJK and Karpas 299 cells four days after infection. Values were normalized to TBP and data were analyzed according to the 2-ΔΔCp method. Results are represented as mRNA levels relative to control. Error bars indicate standard deviation of infected triplicates. (C) Western Blot analysis of C/EBPβ in the three transduced ALK+ ALCL cell lines four days after infection demonstrates successful knockdown. Each lane contained 30 μg protein extract. ALK was used as loading control. (TIF) [file pone.0117780.s001.tif]
