# Supplementary material for: Next-Generation Sequencing Identifies Deregulation of MicroRNAs Involved in Both Innate and Adaptive Immune Response in ALK+ ALCL
Source: PLoS One. 2015 Feb 17;10(2):e0117780. doi: 10.1371/journal.pone.0117780 (PMC4331429; doi:10.1371/journal.pone.0117780)
Supplement: S1 Table — The 82 significantly regulated miRNAs between ALK+ ALCL cell lines and the ALK- cell line and between ALK+ ALCL cell lines and normal T cells are shown and expression (base mean) and significance (padj) values of the significantly regulated miRNAs are indicated. The 56 miRNAs additionally significantly regulated between ALK- ALCL cells and T cells are highlighted in grey. (PDF) [file pone.0117780.s002.pdf]

S1 Table: Significantly regulated miRNAs between ALK+ ALCLs, ALK- ALCLs and normal T cells. The 82 significantly regulated miRNAs between ALK+ ALCL cell lines and the ALK- cell line and between ALK+ ALCL cell lines and normal T cells are shown and expression (base mean) and significance (padj) values of the significantly regulated miRNAs are indicated. The 56 miRNAs additionally significantly regulated between ALK- ALCL cells and T cells are highlighted in grey.

miRNA	ALK- ALCL base mean	ALK+ ALCL base mean	padj	T cells base mean	ALK+ ALCL base mean	padj
hsa-miR-196b	186	0	2,48E-43	7	0	1,37E-08
hsa-miR-155	44475	1205	8,56E-35	3622	1205	1,09E-04
hsa-miR-146a	2525	29	3,34E-27	7082	29	3,03E-37
hsa-miR-203	0	316	1,19E-23	8	316	2,57E-14
hsa-miR-135b	2	405	1,07E-21	6	405	4,46E-17
hsa-miR-135b*	0	119	1,67E-18	0	119	1,60E-17
hsa-miR-503	343	22	1,14E-16	2	22	7,13E-06
hsa-miR-424*	910	78	7,64E-15	28	78	7,90E-03
hsa-miR-182	672	17849	3,79E-14	86	17849	3,24E-27
hsa-miR-183	51	1473	3,79E-14	4	1473	1,11E-27
hsa-miR-542-3p	413	38	1,40E-13	8	38	7,49E-04
hsa-miR-513a-5p	26	0	3,33E-13	1	0	4,78E-02
hsa-miR-450b-5p	1246	134	7,43E-13	19	134	2,00E-06
hsa-miR-3194	28	0	9,89E-13	14	0	2,60E-08
hsa-miR-149	427	51	1,67E-12	3	51	4,80E-08
hsa-miR-193b	1910	240	6,83E-12	73	240	2,34E-03
hsa-miR-199a-5p	28	0	2,28E-11	6	0	9,40E-04
hsa-miR-513c	19	0	2,81E-11	6	0	1,20E-06
hsa-miR-4326	0	30	3,58E-11	9	30	9,93E-03
hsa-miR-574-3p	0	31	3,58E-11	5	31	3,31E-04
hsa-miR-96	2	71	5,09E-11	0	71	1,60E-14
hsa-miR-365	400	55	1,84E-10	17	55	8,50E-03
hsa-miR-450a	438	50	2,94E-10	19	50	1,99E-02
hsa-miR-3200-3p	115	15	1,39E-08	1	15	2,77E-05
hsa-miR-183*	1	34	1,61E-08	0	34	3,85E-10
hsa-miR-9	20	940	5,99E-08	24	940	7,48E-08
hsa-miR-181a-2*	0	38	7,16E-08	615	38	2,66E-08
hsa-miR-33b	304	61	2,74E-07	179	61	5,64E-04
hsa-miR-497	25	2	2,74E-07	9	2	2,26E-03
hsa-miR-548i	0	16	3,78E-06	3	16	2,64E-02
hsa-miR-195	19	1	1,21E-05	8	1	6,18E-03
hsa-miR-9*	1	11	1,89E-04	2	11	5,00E-03
hsa-miR-26a	17721	5467	1,92E-04	200819	5467	9,38E-34
hsa-miR-582-5p	0	6	3,20E-04	17	6	2,53E-02
hsa-miR-20b*	1	22	3,55E-04	0	22	3,23E-05
hsa-miR-1246	57	252	3,62E-04	7	252	4,22E-14
hsa-miR-205	6	0	3,85E-04	2	0	1,80E-02
hsa-miR-223	0	7	8,15E-04	523	7	1,23E-18
hsa-miR-574-5p	9	55	1,12E-03	4	55	9,87E-06
hsa-miR-145	0	59	1,20E-03	0	59	2,34E-03
hsa-miR-34a	1248	443	2,12E-03	42	443	1,05E-08
hsa-miR-335*	0	7	2,45E-03	189	7	4,83E-08
hsa-miR-196a	1772	602	2,47E-03	7	602	2,16E-19
hsa-miR-549	0	49	2,47E-03	0	49	1,72E-03
hsa-miR-301b	651	246	2,82E-03	15	246	3,88E-10

hsa-miR-342-3p	825	300	2,82E-03	15014	300	3,45E-38
hsa-miR-766	80	29	3,37E-03	141	29	5,95E-07
hsa-miR-625*	107	35	3,42E-03	108	35	1,44E-03
hsa-miR-1271	6	1	3,43E-03	7	1	1,04E-03
hsa-miR-3938	0	13	3,49E-03	0	13	1,29E-03
hsa-miR-339-3p	218	720	3,72E-03	2601	720	3,90E-05
hsa-miR-2355-5p	10	2	4,27E-03	17	2	6,01E-05
hsa-miR-181a	60934	23243	7,10E-03	119105	23243	6,47E-08
hsa-miR-874	8	29	7,81E-03	183	29	2,22E-08
hsa-miR-1301	30	10	1,00E-02	35	10	1,44E-03
hsa-miR-625	77	28	1,04E-02	63	28	2,90E-02
hsa-miR-21*	11990	33125	1,11E-02	3020	33125	1,86E-09
hsa-miR-342-5p	54	21	1,11E-02	720	21	1,57E-29
hsa-miR-25*	41	124	1,28E-02	35	124	1,44E-03
hsa-miR-2110	10	3	1,32E-02	16	3	3,52E-04
hsa-miR-221	4000	1627	1,52E-02	3470	1627	2,35E-02
hsa-miR-363	729	6511	1,56E-02	1230	6511	3,33E-02
hsa-miR-92a-2*	0	7	1,56E-02	0	7	6,24E-03
hsa-miR-548n	12	55	1,70E-02	5	55	2,00E-04
hsa-miR-548t	0	3	1,80E-02	0	3	2,66E-02
hsa-miR-194	108	44	1,94E-02	465	44	4,22E-14
hsa-miR-3182	194	4892	1,94E-02	5	4892	6,23E-06
hsa-miR-98	14172	6215	2,43E-02	1375	6215	5,31E-05
hsa-miR-106a	101	668	2,79E-02	31	668	1,88E-04
hsa-miR-192	3599	1669	2,81E-02	15403	1669	3,00E-14
hsa-miR-629*	7	23	2,83E-02	5	23	2,19E-03
hsa-miR-139-5p	1	6	3,19E-02	1	6	3,27E-02
hsa-miR-20b	230	1614	3,19E-02	57	1614	1,20E-04
hsa-miR-942	31	11	3,46E-02	32	11	1,11E-02
hsa-miR-501-3p	62	207	3,48E-02	18	207	3,81E-06
hsa-miR-210	1220	390	3,57E-02	58	390	4,70E-04
hsa-miR-1910	0	8	3,63E-02	0	8	7,91E-03
hsa-miR-320d	1	5	3,88E-02	25	5	3,37E-03
hsa-miR-505	83	37	3,99E-02	84	37	2,19E-02
hsa-miR-145*	0	9	4,03E-02	0	9	3,28E-02
hsa-miR-33b*	35	14	4,37E-02	4	14	2,72E-02
hsa-miR-937	22	8	4,68E-02	1	8	4,62E-03

7
