# Supplementary material for: Next-Generation Sequencing Identifies Deregulation of MicroRNAs Involved in Both Innate and Adaptive Immune Response in ALK+ ALCL
Source: PLoS One. 2015 Feb 17;10(2):e0117780. doi: 10.1371/journal.pone.0117780 (PMC4331429; doi:10.1371/journal.pone.0117780)
Supplement: S2 Table — The expression level tendencies of twelve miRNAs were validated in the three ALK+ ALCL cell lines SUDHL-1, KiJK and Karpas 299, the ALK- ALCL cell line Mac-1 and T cells using RT-qPCR. RT-qPCR values were normalized to miR-106b and data were analyzed according to the 2-ΔΔCp method. NGS results are represented as base mean expression and RT-qPCR results are shown as percentages relative to SUDHL-1 cells (100%). (PDF) [file pone.0117780.s003.pdf]

S2 Table: RT-qPCR validation of miRNA NGS results. The expression level tendencies of twelve miRNAs were validated in the three ALK+ ALCL cell lines SUDHL-1, KiJK and Karpas 299 and the ALK- ALCL cell line Mac-1 and T cells using RT-qPCR. RT-qPCR values were normalized to miR-106b and data were analyzed according to the $2^{-\Delta\Delta Cp}$ method. NGS results are represented as base mean expression and RT-qPCR results are shown as percentages relative to SUDHL-1 cells (100%).

**miRNA**	**hsa-miR-182**		**hsa-miR-183**		**hsa-miR-203**		**hsa-miR-7**	
**method**	**NGS**	**RT-qPCR**	**NGS**	**RT-qPCR**	**NGS**	**RT-qPCR**	**NGS**	**RT-qPCR**
SUDHL-1	12432	100	1412	100	395	100	255	100
KiJK	21425	220	1562	183	352	88	108	50
Karpas 299	19689	244	1446	172	201	51	187	67
Mac-1	672	7	51	7	0	1	132	35
T cells	86	4	4	0	8	5	48	31

**miRNA**	**hsa-miR-146a**		**hsa-miR-146b-5p**		**hsa-miR-181a**		**hsa-miR-181a***	
**method**	**NGS**	**RT-qPCR**	**NGS**	**RT-qPCR**	**NGS**	**RT-qPCR**	**NGS**	**RT-qPCR**
SUDHL-1	37	100	202140	100	5082	100	481	100
KiJK	11	31	61268	28	47126	949	3881	1213
Karpas 299	39	187	1042	0	17521	683	1182	536
Mac-1	2525	4077	3566	2	60934	1198	1539	776
T cells	7082	12014	38699	19	119105	1213	1208	425

**miRNA**	**hsa-miR-181c**		**hsa-miR-29c**		**hsa-miR-342-3p**		**hsa-miR-486-5p**	
**method**	**NGS**	**RT-qPCR**	**NGS**	**RT-qPCR**	**NGS**	**RT-qPCR**	**NGS**	**RT-qPCR**
SUDHL-1	5	100	283	100	448	100	16	100
KiJK	62	1008	1155	177	198	56	5	116
Karpas 299	299	713	756	208	252	93	2106	151
Mac-1	283	1193	480	312	825	229	788	200
T cells	3493	1188	12645	3002	15014	4142	2915	221
