# Supplementary material for: Next-Generation Sequencing Identifies Deregulation of MicroRNAs Involved in Both Innate and Adaptive Immune Response in ALK+ ALCL
Source: PLoS One. 2015 Feb 17;10(2):e0117780. doi: 10.1371/journal.pone.0117780 (PMC4331429; doi:10.1371/journal.pone.0117780)
Supplement: S3 Table — The 80 significantly regulated miRNAs in at least one of the analyzed ALK+ ALCL cell lines by C/EBPβ are shown and miRNA expression levels (base mean of triplicates) of the three ALK+ ALCL cell lines SUDHL-1, KiJK and Karpas 299 with (pF-C/EBPβ) and without (pF) C/EBPβ knockdown as well as the ALK- ALCL cell line Mac-1 and normal T cells are depicted. (PDF) [file pone.0117780.s004.pdf]

S3 Table: Significantly regulated miRNAs after C/EBPβ knockdown. The 80 significantly regulated miRNAs in at least one of the analyzed ALK+ ALCL cell lines by C/EBPβ are shown and miRNA expression levels (base mean of triplicates) of the three ALK+ ALCL cell lines SUDHL-1, KiJK and Karpas 299 with (pF-C/EBPβ) and without (pF) C/EBPβ knockdown as well as the ALK- ALCL cell line Mac-1 and normal T cells are depicted.

ID	SUDHL pF base mean	SUDHL-1 pF-C/EBPβ base mean	KiJK pF base mean	KiJK pF-C/EBPβ base mean	Karpas 299 pF base mean	Karpas 299 pF-C/EBPβ base mean	Mac-1 base mean	T cells base mean
hsa-let-7d	543	356	2354	1867	1726	1456	4207	1914
hsa-let-7g	6659	4697	8158	7374	11530	13592	11558	92352
hsa-let-7i	3202	1953	5851	5203	4637	4817	6044	16751
hsa-miR-101*	9	21	15	15	14	17	5	61
hsa-miR-103	9943	9206	5233	4054	5611	5359	8770	5393
hsa-miR-106a	1228	799	1	1	776	671	101	31
hsa-miR-106b*	391	276	393	337	363	352	432	342
hsa-miR-10a	496	747	38	53	904	1029	267	4737
hsa-miR-1246	133	465	388	737	235	296	57	7
hsa-miR-1248	3	10	14	13	6	14	2	5
hsa-miR-1261	35	19	20	23	22	23	9	9
hsa-miR-1274b	8944	2889	7522	5730	7445	8482	3300	3565
hsa-miR-128	773	1091	926	1015	938	689	621	901
hsa-miR-1291	412	132	118	118	220	95	132	114
hsa-miR-1293	3	3	1	6	0	0	0	0
hsa-miR-135b*	155	113	124	115	79	72	0	0
hsa-miR-142-3p	5689	7820	5473	6745	4745	4616	9212	27953
hsa-miR-143	630	242	7	3	62	31	19	152
hsa-miR-1468	11	4	41	34	6	6	20	62
hsa-miR-146a	37	17	11	8	39	61	2525	7082
hsa-miR-146b-3p	6213	2382	1866	1354	45	29	88	2048
hsa-miR-146b-5p	202140	76407	61268	42117	1042	720	3566	38699
hsa-miR-148a*	676	586	2591	1823	90	64	197	113
hsa-miR-155	3608	2128	2	1	4	7	44475	3622
hsa-miR-181a	5082	7140	47126	62767	17521	26417	60934	119105
hsa-miR-181a*	481	1088	3881	5949	1182	1962	1539	1208
hsa-miR-181b	1043	1868	9727	12252	3201	5067	7133	4238
hsa-miR-181c	5	12	62	115	299	283	283	3493
hsa-miR-18b	225	151	1	1	116	69	20	4
hsa-miR-193a-5p	83	40	31	16	45	50	36	13
hsa-miR-193b	336	189	0	0	382	169	1910	73
hsa-miR-203	395	213	352	159	201	100	0	8
hsa-miR-204	5	12	1	0	1	0	0	0
hsa-miR-20b	3152	2057	1	1	1690	1632	230	57
hsa-miR-22	35466	34625	4454	6404	3747	3772	8935	25356
hsa-miR-221*	27	40	24	19	47	80	54	10
hsa-miR-222	1979	1831	1740	1133	2285	3237	3298	1040
hsa-miR-222*	7	22	16	11	13	11	49	5
hsa-miR-223	19	4	3	1	0	1	0	523
hsa-miR-223*	11	1	3	0	0	0	0	16
hsa-miR-26a	8577	8070	2034	2883	5792	5703	17721	200819
hsa-miR-26b	5991	3766	3034	2696	3939	3325	6891	31932
hsa-miR-27a	2293	2378	2959	4561	5959	5365	6889	10857
hsa-miR-29a	1663	1982	2049	2577	4607	4530	4248	36185

hsa-miR-29b-1*	24	14	32	15	50	50	25	23
hsa-miR-29c	283	386	1155	1766	756	699	480	12645
hsa-miR-3168	54	101	70	69	65	120	67	44
hsa-miR-3182	2278	2507	11888	21342	511	1975	194	5
hsa-miR-3195	25	15	29	93	9	12	45	24
hsa-miR-33a*	15	19	13	28	32	31	34	56
hsa-miR-340	1398	1198	1570	1244	1647	1359	0	1093
hsa-miR-345	1206	960	237	161	1015	658	663	162
hsa-miR-3605-3p	18	7	9	3	2	6	10	12
hsa-miR-3605-5p	5	2	1	4	1	2	2	0
hsa-miR-3607-3p	165	254	213	315	176	128	114	1721
hsa-miR-3607-5p	40	25	24	21	38	75	17	304
hsa-miR-361-5p	187	257	143	204	210	200	114	575
hsa-miR-363	12032	7537	5	5	7497	5759	729	1230
hsa-miR-3647-3p	12	28	33	39	56	57	32	307
hsa-miR-365	88	42	5	5	72	33	400	17
hsa-miR-3681	0	3	10	9	2	2	0	0
hsa-miR-378*	33	53	16	20	22	28	21	14
hsa-miR-4301	69	161	2034	5924	218	301	261	198
hsa-miR-449a	20	40	1	1	1	1	3	0
hsa-miR-449c	29	71	1	1	2	3	7	0
hsa-miR-486-5p	16	40	5	8	2106	3028	788	2915
hsa-miR-501-3p	358	432	189	101	73	72	62	18
hsa-miR-502-3p	1042	986	549	414	385	416	294	122
hsa-miR-548u	4	4	8	1	3	4	1	1
hsa-miR-551b	7	10	95	134	110	84	63	0
hsa-miR-615-3p	16	28	298	296	53	40	214	1
hsa-miR-625	23	32	32	68	29	30	77	63
hsa-miR-625*	26	22	28	51	51	62	107	108
hsa-miR-7	255	164	108	72	187	187	132	48
hsa-miR-708	1	1	804	1126	683	952	907	1
hsa-miR-744	302	197	255	218	135	78	378	100
hsa-miR-942	8	17	8	19	17	22	31	32
hsa-miR-98	2167	1550	10260	7920	6218	5197	14172	1375
hsa-miR-99a	92	131	0	0	0	0	6	186
hsa-miR-99a*	10	17	0	0	0	0	0	13
