# Supplementary material for: Next-Generation Sequencing Identifies Deregulation of MicroRNAs Involved in Both Innate and Adaptive Immune Response in ALK+ ALCL
Source: PLoS One. 2015 Feb 17;10(2):e0117780. doi: 10.1371/journal.pone.0117780 (PMC4331429; doi:10.1371/journal.pone.0117780)
Supplement: S4 Table — Comparison of our data with two previously published miRNA profiles of ALK+ ALCL by Merkel et al. [29] and Liu et al. [31]. Shown are the miRNAs, which were found in at least two different studies associated with ALK+ ALCLs. In the studies different cell lines and/ or tumor specimens were used. X indicates, that miRNAs are differentially regulated by ALK and x* symbolizes, that miRNAs are associated with ALK+ ALCL. (PDF) [file pone.0117780.s005.pdf]

S4 Table: Comparison of significantly ALK+ ALCL associated miRNAs between different studies [29, 31]. Comparison of our data with two previously published miRNA profiles of ALK+ ALCL by Merkel et al. [29] and Liu et al. [31]. Shown are the miRNAs, which were found in at least two different studies associated with ALK+ ALCLs. In the studies different cell lines and/ or tumor specimens were used. X indicates, that miRNAs are differentially regulated by ALK and x* symbolizes, that miRNAs are associated with ALK+ ALCL.

	Our data	Merkel et al.	Liu et al.	
miRNA	SUDHL-1, KiJK, Karpas 299 / Mac-1	Karpas 299, SR-786, SUDHL-1/ Mac2a, FEPD and tumorspecimen	SupM2/TS (ALK knockdown)	tumor specimen
hsa-miR-106a	x	x	x	x
hsa-miR-20b	x	x	x	x
hsa-miR-363	x	x*	x	x
hsa-miR-17	x*	x	x	x
hsa-miR-93	x*	x	x	x
hsa-let-7d	x		x	x
hsa-miR-132	x		x	x
hsa-miR-505	x		x	x
hsa-miR-145	x		x	x
hsa-miR-18b	x*		x	x
hsa-miR-155	x	x		x*
hsa-miR-20a	x*	x	x	
hsa-miR-146a	x	x*		x*
hsa-miR-101	x*	x*	x	
hsa-miR-34a	x		x	
hsa-miR-146b-5p	x	x*		
hsa-miR-365	x		x	
hsa-miR-181a	x		x	
hsa-miR-193b	x		x	
hsa-miR-221	x		x	
hsa-miR-223	x		x	
hsa-miR-98	x		x	
hsa-miR-135b	x			x*
hsa-miR-886-3p		x		x*
hsa-miR-708	x*			x*
hsa-miR-886-5p		x*		x*
